# Natural Materials for 3D Printing and Their Applications

**DOI:** 10.3390/gels8110748

**Published:** 2022-11-17

**Authors:** Chunyu Su, Yutong Chen, Shujing Tian, Chunxiu Lu, Qizhuang Lv

**Affiliations:** 1 College of Biology & Pharmacy, Yulin Normal University, Yulin 537000, China; 2 Guangxi Key Laboratory of Agricultural Resources Chemistry and Biotechnology, Yulin 537000, China

**Keywords:** 3D printing, natural materials, bio-ink, composite scaffold, tissue engineering applications

## Abstract

In recent years, 3D printing has gradually become a well-known new topic and a research hotspot. At the same time, the advent of 3D printing is inseparable from the preparation of bio-ink. Natural materials have the advantages of low toxicity or even non-toxicity, there being abundant raw materials, easy processing and modification, excellent mechanical properties, good biocompatibility, and high cell activity, making them very suitable for the preparation of bio-ink. With the help of 3D printing technology, the prepared materials and scaffolds can be widely used in tissue engineering and other fields. Firstly, we introduce the natural materials and their properties for 3D printing and summarize the physical and chemical properties of these natural materials and their applications in tissue engineering after modification. Secondly, we discuss the modification methods used for 3D printing materials, including physical, chemical, and protein self-assembly methods. We also discuss the method of 3D printing. Then, we summarize the application of natural materials for 3D printing in tissue engineering, skin tissue, cartilage tissue, bone tissue, and vascular tissue. Finally, we also express some views on the research and application of these natural materials.

## 1. Introduction

Three-dimensional bioprinting technology, also known as 3D printing, is a popular technology used among materials scientists and biologists. Three-dimensional printing technology is widely used in tissue materials and related fields because of its rapidity, strong compatibility, and short time consumption. Three-dimensional printing has a wide range of applications, not only for the rapid printing of building materials [1], but also for the repair of missing organs or tissues [2,3]. Three-dimensional printing usually uses non-toxic and harmless raw materials to produce the objects we need, or to produce some biological tissues for repairing tissue wounds. Due to advances in regenerative medicine and tissue engineering, and with the help of 3D bioprinting technology, it is possible to repair damaged organs or to regenerate tissues. Therefore, 3D bioprinting technology has great medical value and application value in tissue engineering and regenerative medicine.

Three-dimensional printing cannot be performed without the use of bio-ink. The sources of bio-inks can be divided into synthetic material bio-inks and natural material bio-inks. The bio-inks prepared using synthetic materials come in a variety of types and different shapes, and can be well controlled to obtain some required characteristics. They are widely used in 3D printing, but the production process is usually complex, with high requirements for equipment and a high cost. Natural material bio-inks can be very good for overcoming these problems: the raw natural material is abundant, easy to process and modify, has excellent mechanical properties, high plasticity, good biocompatibility, and high cell activity; it is very suitable for the preparation of biological ink, and makes itself useful in the field of tissue engineering and so on, with a very extensive application [4]. For example, bio-ink, a natural material based on chitosan, has a good immune response and still shows excellent bioactivity and biocompatibility after modification, making is very useful for 3D printing [5]. Pectin can play an important role in bone tissue engineering via 3D printing due to its non-toxicity, water retention, and solubility [6]. Silk fibroin bio-ink can be 3D printed to produce composites that can be used in tissue engineering [7]. There are many examples of natural materials such as these that are used for 3D printing in fields such as tissue engineering, which will be mentioned in this paper.

At present, research on 3D printed natural materials is still being carried out, and the practical applications of natural materials that are found and reported in related fields are also becoming more and more abundant. Therefore, the purpose of this review is to describe the natural materials that are used in 3D printing, and the scientific knowledge of the natural materials, as well as summarizing the current research and application contents of the materials. Especially from the perspective of tissue engineering and other fields, we emphasize the application of 3D-printed natural materials in tissue engineering and other fields. Firstly, we introduce the natural materials used in 3D printing and their properties, and summarize the physical and chemical properties of these natural materials, and their applications in tissue engineering after modification. Secondly, we focus on the modification methods used for 3D printing materials, including physical methods, chemical methods, and protein self-assembly methods. We also discuss the method of 3D printing. Then, we summarize the application of natural materials for 3D printing in tissue engineering, skin tissue, cartilage tissue, bone tissue, and vascular tissue. Finally, we also express some of our views on the research and application of natural materials for 3D printing. The purpose of this review is to provide an overview of the current research progress of natural materials for 3D printing, with special attention toward their applications in tissue engineering, skin tissue, cartilage tissue, bone tissue, and vascular tissue.

## 2. Classification of Natural Biomaterials

Natural materials for 3D printing are a hot topic of research, and they come from animals, plants, and microbes. The existence of these natural materials has a very important influence on the homeostasis of the internal environment, the maintenance of the normal level of cells, and the regulation of various functions of the body. Additionally, the natural materials used for 3D printing have the advantages of easy processing and modification, abundant raw materials, excellent mechanical properties, good biocompatibility, high plasticity, high biodegradability, and high cell activity, which makes them very suitable for the preparation of biological ink and use in 3D printing. This study divides these natural materials into three categories—proteins, polysaccharides, and other categories—each of which contain the corresponding natural materials. Although there are significant differences between different kinds of natural materials, after the heating method, physical method, chemical method, and protein self-assembly method of modification, the prepared bio-ink or composite materials and stents can be effectively applied in tissue engineering, skin tissue, cartilage tissue, bone tissue, and vascular tissue, and these contents will be mentioned in this review [8].

### 2.1. Protein Class

Proteins are macromolecules whose amino acids condense through dehydration to form a polypeptide chain, and then they wind and fold them to form a three-dimensional spatial structure. Protein is an indispensable component of human cells and tissues, and it plays a very important role in human life activities. Many proteins have many good characteristics, such as biocompatibility, easy chemical repair, and biodegradability, which greatly improve the protein research and application scope; the protein after processing into bio-ink and biological scaffolds can be effectively applied in tissue engineering, skin engineering, cartilage tissue, bone tissue, and vascular tissue. Common natural proteins are: platelet lysate, collagen, gelatin, silk fibroin, albumin, fibrin, elastin, and keratin.

#### 2.1.1. Platelet Lysate

Platelet lysate (PL) is a natural protein that is purified and extracted from platelet-rich plasma. PL contain many different kinds of cytohormones, cell growth factors, and a variety of proteins. Through the processing and modification of the PL, the prepared bio-ink can be used for vascular regeneration and in vitro tissue development [9]. For example, Babrnakova et al. used bovine platelet lysate (BPL) to modify the collagen–polysaccharide scaffold for modification, and the results showed that the scaffold, after being modified using BPL, showed better cell compatibility and cell activity, and could effectively promote vascular regeneration and wound healing, which can be applied to trauma repair and other fields [10]. Mendes et al. prepared a PL nano-ink with PL hydrogel modified with cellulose nanocrystals. After 3D printing, this material still had long-term cellular activity, and could effectively promote cell growth, proliferation, and differentiation, which is suitable for the development of 3D tissues in vitro [11].

#### 2.1.2. Collagen

**Figure 1:**
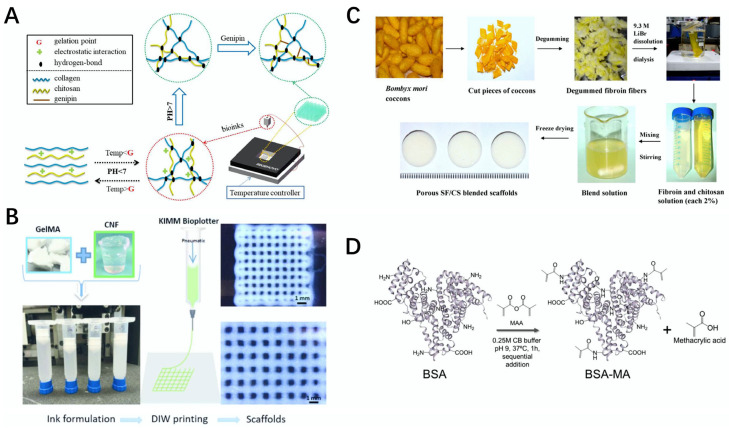
Schematic diagram of the preparation of some protein bioscaffolds and bio-inks. (**A**) Schematic representation of the 3D printed Col–chitosan scaffold [15]. (**B**) Schematic diagram of the preparation of CNF/GelMA bio-inks and their use for 3D printing into scaffolds [16]. (**C**) Schematic diagram of the preparation of the SF–chitosan composite scaffold [17]. (**D**) Schematic diagram of the preparation of the BSA–MA hydrogel [18].

Collagen (Col) is an important component of animal connective tissue, and it is also the most abundant class of proteins in mammals. Collagen usually appears as a white or transparent powder with good ductility, biodegradability, biocompatibility, and water retention, which has been widely used in food, tissue engineering, and medicine [12,13]. Col was used to make bio-ink and is used for 3D printing, and the bio-scaffolds prepared using Col are widely used in tissue engineering. For example, Turk et al. used chitosan, functionalized multiwalled carbon nanotube (f-MWCNT), hydroxyapatite, and Col to prepare a Col/f-MWCNT/hydroxyapatite scaffold using freeze-drying technology; the composite bioscaffold has compressive resistance, high bioactivity, and good biocompatibility, and can be applied to bone tissue and other fields [14]. Suo et al. used Col and chitosan as materials to prepare a Col–chitosan composite bio-ink, and then printed the Col–chitosan composite scaffold with 3D printing technology; the scaffold had a stable structure, and outstanding mechanical properties and non-toxic properties, and it effectively enhanced cell proliferation, with promising applications in tissue engineering and other fields (Figure 1A) [15].

#### 2.1.3. Gelatin

Gelatin is the product of collagen denaturation, and its molecule is a helix formed from three polypeptide chains intertwined with each other. Gelatin usually appears as a pale yellow or colorless powder solid, which can be hydrolyzed to obtain a variety of amino acids. Gelatin usually appears as a light yellow or colorless powder solid, after which hydrolysis can be used to obtain multiple amino acids. Due to its good biocompatibility, low cost, and easy processing properties, the prepared bio-ink painting is widely used in tissue engineering and other fields [19,20]. For example, Wang et al. modified gelatin with heparin to prepare gelatin–heparin fiber material, which showed good biological activity, and at the same time, the material could achieve a local sustainable release of heparin for more than 14 days, which could be used as a controlled drug delivery device for continuous drug release in vascular tissues [21]. Methylacryloylated gelatin (GelMA) is a kind of gelatin that is widely used and processed into bio-ink to prepare stents, which can be used for trauma repair. Xu et al. prepared a CNF/GelMA bio-ink from GelMA and cellulose nanofibril (CNF), and 3D printed a bio-scaffold with direct ink writing (DIW). The scaffold showed good void fraction, and low toxicity and biocompatibility; at the same time, this scaffold could effectively promote the proliferation of fiber cells, which can be applied in wound repair and other fields (Figure 1B) [16].

#### 2.1.4. Silk Fibroin

Silk fibroin (SF) is a natural protein derived from silk with good biocompatibility, tensile resistance, and flexibility. Silk fibroin is modified using other materials to make bio-ink or biological materials, which can be used in the development of tissues and organs [22,23]. For example, Kim et al. modified SF with glycidyl methacrylate (GMA), developed a SF-GMA bio-ink, and prepared a SF-GMA hydrogel with digital light processing (DLP) 3D printing. This printing material has good biocompatibility and stability, and can be applied to the development of tissues and organs, as required [24]. Bhardwaj et al. prepared a SF–chitosan composite scaffold using a freeze-drying method with SF and chitosan as the materials, which had good compression resistance, antibacterial properties, and biocompatibility, and it was also concluded from in vitro experiments that the scaffold could effectively promote the adhesion, growth, and proliferation of feline fibroblasts, and could be used as a potential scaffold for tissue engineering (Figure 1C) [17].

#### 2.1.5. Albumin

Albumin is the main protein in human plasma, and is used to maintain the stability of plasma colloid osmotic pressure, which is very important for human health. Albumin shows good solubility and good biocompatibility, and it can detoxify the poisoning caused by heavy metals. Biological scaffolds or composite materials prepared through the interaction of albumin with other materials are widely used in the field of cartilage tissue [25,26]. Lyu et al. modified a polyethylene oxide/chitin/chitosan scaffold with albumin, and through culture experiments with porcine knee chondrocytes, high concentrations of albumin effectively promoted the adhesion, proliferation, and differentiation of this cell, and facilitate the growth and development of chondrocytes, which can be applied in the field of cartilage tissue [27]. Ferracci et al. prepared a photocurable bovine serum albumin methacryloyl (BSA-MA) hydrogel with bovine serum albumin (BSA) and methacrylic anhydride (MAA) as the raw materials, and modified it with carbonate–bicarbonate (CB). The hydrogel had good cell activity, mechanical properties, and biocompatibility, and at the same time, the hydrogel effectively supported the growth and proliferation of cells, and can be used in 3D printing and tissue engineering (Figure 1D) [18].

#### 2.1.6. Fibrin

**Figure 2:**
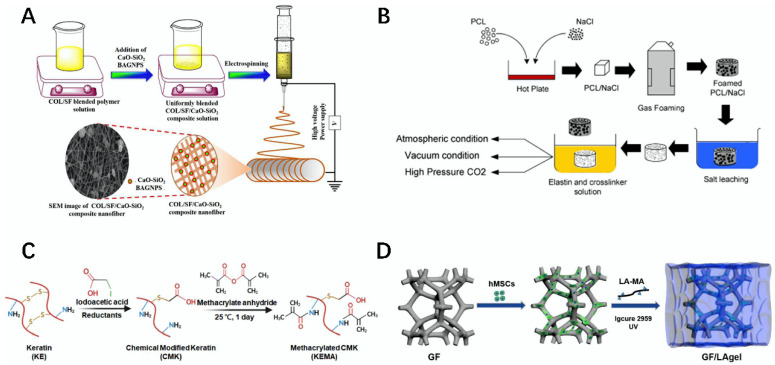
Schematic diagram of the preparation of biocomposite scaffolds and bio-inks using proteinaceous natural materials. (**A**) Schematic diagram of the preparation of the Col/SF/CaO-SiO_2_ composite fibers [31]. (**B**) Schematic diagram of the preparation of the PCL/elastin composite scaffold [32]. (**C**) Schematic diagram of the preparation of the KEMA bio-ink [33]. (**D**) Schematic diagram of the preparation of the GF/LAgel composite scaffold [34].

Fibrin is an important protein that plays a role in hemostasis in the blood. It is highly insoluble in water, but provides mechanical strength to the organism and plays a protective role. Fibrin is highly biodegradable and biocompatible, and can be used for trauma repair and tissue engineering [28,29]. Zhao et al. first prepared a PLLA scaffold with poly (L-Lactide) (PLLA) and other materials, and then made a PLLA/fibrin gel composite biological scaffold with fibrin gel modification; the scaffold had good cytocompatibility, biological activity, and good mechanical properties; at the same time, it could effectively promote the growth and proliferation of rabbit auricular chondrocyte; this is suitable for cartilage tissue repair and it can be used as a potential material [30]. Wu et al. first used Col, CaO-SiO_2_, and SF as the materials to prepare a Col/SF/CaO-SiO_2_ composite fiber through a sol–gel process and electrospinning The material showed good thermal stability, biodegradability, and biocompatibility. At the same time, the material could accelerate the growth and development of blood vessels, and the formation of bone tissue, which is suitable for the repair of bone defects (Figure 2A) [31].

#### 2.1.7. Elastin

Elastin is the major component of elastic fiber in dermal tissues. Elastin usually shows light yellow acid–base resistance, and its presence plays an important role in skin elasticity. As an important natural protein, elastin is modified to prepare bio-inks, which can be used in the field of tissue engineering after 3D printing [35]. For example, Dai et al. developed an elastin-like polypeptide (ELP) bio-ink; the material combined with collagen I improved cell viscosity and exhibited good cell activity, biocompatibility, and mechanical properties that can be used as a potential bio-ink for 3D printing [36]. Annabi et al. used poly (ε-caprolactone) (PCL) and NaCl as the materials to prepare a PCL/elastin-porous composite scaffold through a series of processing modifications. The prepared stent had high porosity, a high water absorption rate, and good biocompatibility and mechanical properties, and could be applied in the field of tissue engineering (Figure 2B) [32].

#### 2.1.8. Keratin

Keratin (KE) is the main protein that constitutes the fur hair follicle and the raw skin epidermis. KE is insoluble in water, but has good mechanical strength and stability. After modification or compositing, the prepared composite scaffold and bio-ink produced can be well used in the tissue field [37,38]. Nayak et al. prepared a KE/agar composite scaffold from KE and agar using a freeze extraction technique, and this scaffold exhibited good mechanical strength and cellular activity. At the same time, the scaffold showed good biocompatibility for the myofibroblast cell line, and could effectively promote the proliferation of the cell, which can be used for trauma repair and skin regeneration [39]. Yu et al. used KE as the material, through modification via lodoacetic acid and methacrylate anhydride, and prepared a keratin methacrylate (KEMA) composite bio-ink, which showed good bioactivity and cytocompatibility, and showed good potential in 3D bioprinting as a potential material for tissue engineering (Figure 2C) [33].

### 2.2. Polysaccharide

Polysaccharides are polymer carbohydrates formed via the combination of monosaccharides. They are generally insoluble in water, but the corresponding monosaccharides can be formed after hydrolysis. At the same time, polysaccharide is also a very important class of substances in nature, which is even more indispensable for living organisms. Additionally, because polysaccharides have many excellent characteristics, such as biocompatibility, low price, antibacterial, and easy chemical modification, these greatly improve their application range. Polysaccharides are processed into composite materials, scaffolds, and bio-inks. After 3D bioprinting, they can be effectively used in tissue engineering, skin engineering, cartilage tissue, bone tissue, and vascular tissue [40]. Common natural polysaccharides are laminarin, agarose, sodium alginate, hyaluronic acid, chitosan, chondroitin sulfate, carrageenan, xanthan gum, heparin, fucoidan, guar gum, and pectin.

#### 2.2.1. Laminarin

Laminarin is a low-molecular polysaccharide that is present in fucoidan, and it is linked using glycosidic bonds at (1,6) and β-glucans linked at (1,3). Due to its biological properties such as non-toxicity, antibacterial, biodegradability, and biocompatibility, it has attracted much attention in wound repair, anti-inflammatory uses, and anti-oxidation. After modification, it can be used in tissue engineering and other fields [41,42]. For example, Amaral et al. prepared bio-inks by combining alginate and boronic acid-functionalized laminarin with divalent cations. The ink had excellent mechanical properties and suitable rheological properties to serve as a culture matrix for different cell types. The hydrogels prepared via 3D printing using this bio-ink can be used for the sustainable culture of cells [43]. Lin et al. first interacted with graphene foam (GF) and laminarin hydrogel (LAgel) to prepare a GF/LAgel composite scaffold under ultraviolet light (UV) irradiation. The composite scaffold had better toughness, cell adhesion, and regulation of cell behavior, but could also very effectively enhance the cell diffusion and adhesion of human mesenchymal stem cells (hMSCs), with a very promising application prospect in the field of tissue regeneration (Figure 2D) [34].

#### 2.2.2. Agarose

**Figure 3:**
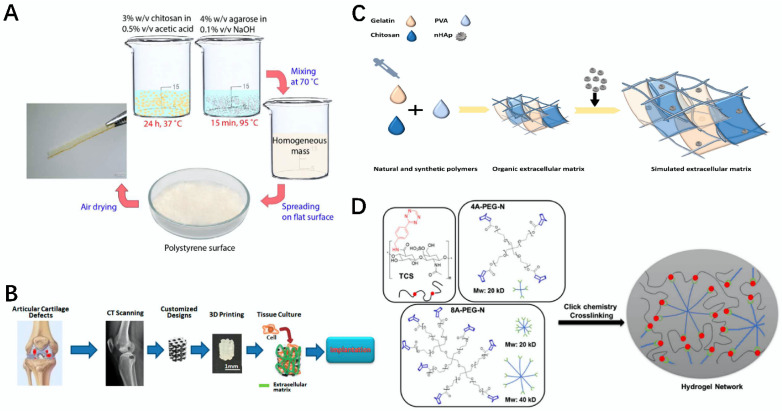
Schematic diagram for the preparation of bio-scaffolds, composites, and hydrogels using polysaccharides. (**A**) Schematic diagram of the preparation of the chitosan–agarose membrane as a skin surrogate [47]. (**B**) Schematic diagram of the water-based light-cured PU/HA scaffolds preparation [48]. (**C**) Schematic diagram of the preparation of the gelatin/chitosan/PVA/nHAp (GCPH) composite scaffold [49]. (**D**) Schematic diagram of the preparation of the TCS-A-PEG-N porous hydrogel network [50].

Agarose (AG) is a polysaccharide derived from red algae, whose basic structure is 1,4-linked 3,6-anhydro-α-L-galactose and a 1,3-linked β-D-galactose alternating long-chain [44]. Due to its biocompatibility, non-toxicity, and adjustable characteristics, AG can be well applied in vascular regeneration and skin repair [45]. For example, a composite hydrogel was prepared by Kreimendahl et al. via 3D bioprinting, using agarose fibrinogen and agarose type I collagen. It was able to promote the formation of a capillary network structure, and to some extent, induced vascularization [46]. On the other hand, Vivcharenko et al. prepared a non-toxic, degradable, elastic, and cell-growth-friendly chitosan/agarose film material using chitosan and AG as materials, using a process such as heating to mix the materials, followed by cooling and stirring, which can be well used as a substitute for artificial skin (Figure 3A) [47].

#### 2.2.3. Sodium Alginate

Sodium alginate (SA) is a natural polysaccharide that is often processed into bio-ink for 3D bioprinting. SA is composed of α-L-guluronic acids and (1–4) linked β-D-mannuronic, which is deeply favored by its own biodegradation, biocompatibility, non-toxicity, and non-immunogenicity. Additionally, SA can be obtained from the cell walls of cheap fucoidan such as kelp, so it is often processed into bio-ink for 3D bioprinting [51,52]. For example, Saini et al. prepared a gelatin-SA-PVP three-dimensional biological scaffold using a freeze-drying method with gelatin, SA, and poly (vinyl alcohol) (PVP) as the raw materials, which exhibited good mechanical properties, non-cytotoxicity, antibacterial properties, and compression resistance, enabling it for good application in the field of bone tissue [53]. Seyedmahmoud et al. used GelMA and SA to prepare a GelMA-SA bio-ink under the action of CaCl2 and UV light, which could be prepared into a composite material after 3D printing, and it could effectively improve the activity of cell metabolism. Therefore, it is a very promising bio-ink in tissue regeneration [54].

#### 2.2.4. Hyaluronic Acid

Hyaluronan (HA) is a natural glycosaminoglycan formed by the alternating junctions of D-glucuronic acid and D-N-acetylglucosamine, and it generally exists in animal joint fluid, cartilage, and eye vitreous. It is an important part of the human extracellular matrix, has a high solubility and excellent water absorption ability, and it shows a good effect in lubrication, buffer, tolerance, and water [55,56]. At the same time, it is processed into 3D printed bio-ink, and the composite scaffolds, prepared via 3D printing technology, have a wide and effective application in fields such as cartilage engineering [57]. For example, Kenar et al. used poly (L-lactide-co-ε-caprolactone)/collagen and HA to prepare a composite fiber scaffold. It showed good water absorption, support, and cell adhesion, which could better induce vascular formation and promote the formation of a vascular network [58]. Shie et al. prepared water-based light-cured PU/HA scaffolds via 3D printing using polyurethane (PU) and HA as the base materials. The scaffold exhibits the excellent characteristics of non-toxicity, cytocompatibility, and a high printing resolution, which can effectively promote cell proliferation and accelerate the regeneration of damaged cartilage; it is well suited for the repair of different kinds of cartilage damage, and has very good prospects for applications in cartilage repair (Figure 3B) [48].

#### 2.2.5. Chitosan

Chitosan is a natural polysaccharide composed of two repeating units, N-acetyl-D-glucosamine and D-glucosamine. It is not often present in natural environments, but it needs to be obtained via the deacetylation of chitin. Chitosan is also the only positively charged alkaline polysaccharide found so far. Chitosan has the excellent characteristics of being biodegradable, biocompatible, and antibacterial; its chemical modification is easy, and it can effectively promote wound healing. It is prepared into a composite bio-ink, and after 3D printing, the prepared material plays a very good application in skin trauma repair [59,60]. For example, He et al. mixed chitosan modified with methacryloyl groups (CHIMA) and acrylamide (AM) into a new brand chitosan–AM bio-ink, and then 3D bioprinting based on digital light processing (DLP) was used to produce a hydrogel with good mechanical properties and adjustable cell compatibility. This bio-ink is very suitable for 3D bioprinting, and it has a very promising prospect for application in the construction of tissue-engineered organs [61]. Using chitosan’s ease of processing and its other characteristics, biological scaffold prepared by it also has good application in the field of bone tissue [62]. Ma et al. prepared a gelatin/chitosan/PVA/nHAp (GCPH) composite scaffold using gelatin, chitosan, polyvinyl alcohol (PVP), and nanohydroxyapatite (nHAp). This scaffold can be used to simulate the composition and structure of bone, but it also can effectively promote cell proliferation and improve the attachment ability of cells, so that it is widely used in the field of bone tissue (Figure 3C) [49].

#### 2.2.6. Chondroitin Sulfate

Chondroitin sulfate (CS) is an acidic mucopolysaccharide extracted from animal cartilage tissue; it is composed of D-glucuronic acid and N-acetylgalactose, and has high water solubility, but is insoluble in organic solvents. Additionally, under acid conditions, it will be hydrolyzed to generate glucuronic acid and amino hexose, and for pain relief, promote cartilage regeneration and improve the effects of arthritis [63]. Bio-ink processing from CS is widely used in 3D bioprinting, and it can be used in fields such as cartilage tissue and vascular tissue [64,65]. For example, a bio-ink of cellulose–SA was developed by Lafuente-Merchan et al. Then, they added the CS component to it. The test results showed that the CS addition could effectively improve the characteristics of the cellular activity and rheology of the bio-ink. Simultaneously, the biological scaffold prepared using sodium cellulose-alginate–CS can effectively promote cartilage differentiation, providing a new method for cartilage regeneration [66]. Sousa et al. used Tetrazine–CS (TCS), 8-Arm polyethylene glycol-Norbornene (8A-PEG-N), and 4-Arm polyethylene glycol-Norbornene (4A-PEG-N) as materials to prepare TCS-A-PEG-N porous hydrogels. The gel had stable mechanical properties, mechanical properties, and adjustable rheology, and its biocompatibility was also very good. At the same time, it effectively increased the number of transport blood vessels and enhanced blood irrigation, providing a new strategy for trauma repair and skin tissue repair in situ (Figure 3D) [50].

#### 2.2.7. Carrageenan

**Figure 4:**
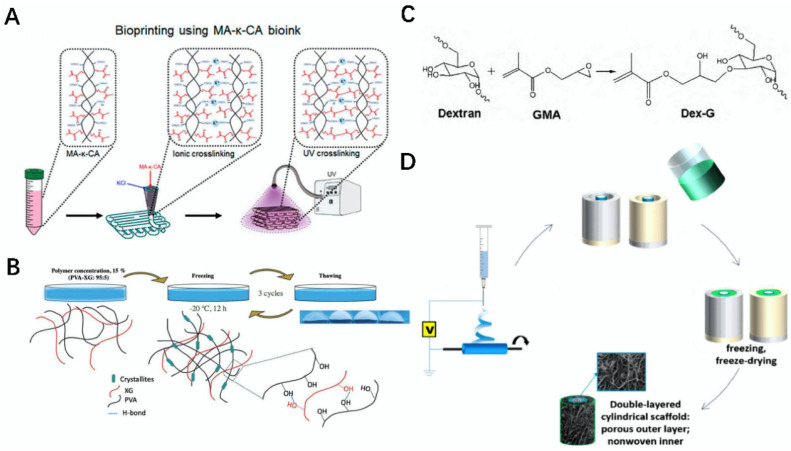
Schematic diagram of the preparation of related polysaccharide bio-inks and bioscaffolds. (**A**) Bioprinting of MA-k-CA bio-ink [70]. (**B**) Schematic diagram of preparation of PVA-XG hydrogel [71]. (**C**) Schematic diagram of preparation of Dex-G glucose-sensitive hydrogel [72]. (**D**) Schematic diagram of preparation of a double-layer tubular biological scaffold modified by heparin [73].

Carrageenan (CRG) is a natural polysaccharide extracted from red algae sea grass, generally white or light brown particles or powder dissolved in hot water to form a transparent or milky white solution. According to the different binding forms of sulfate in CRG, it can be divided into β-type, μ-type and κ-type, γ-type, and λ-type, etc. CRG itself has the advantages of good biocompatibility, good stability, and excellent anticoagulation, so it has a very good research application in tissue engineering and other fields [67]. Carrageenan is used in the preparation of biological scaffolds or hydrogels, and the resulting materials can be used in bone tissue and other tissue engineering fields [68]. For example, Loukelis et al. used κ-carrageenan, chitosan, gelatin, and KCl to cross-link to form a three-dimensional biological scaffold, which had a porosity of more than 80%, good biocompatibility and a strong structure. Moreover, all components of the scaffold were conducive to bone differentiation, which has broad development prospects in bone tissue repair [69]. Lim et al. prepared a MA-k-CA bio-ink on the basis of which a MA-k-CA hydrogel could be prepared using the dual cross-linking of ions and ultraviolet. The bio-ink had adjustable mechanical properties and rheological properties. At the same time, the bio-ink had outstanding mechanical properties, a high degree of biological activity, and a wide range of applicable temperature, and it can be used as a wide range of bio-inks in the field of tissue engineering (Figure 4A) [70].

#### 2.2.8. Xanthan Gum

Xanthan gum (XG) is a microbial polysaccharide widely used in the fields of bone tissue and tissue engineering, and it is a light yellow or white powder at room temperature. XG itself has good water solubility, thermal stability, acid-base resistance, biocompatibility, and unique rheological properties. According to these properties, XG can be made into composite materials via interaction with other materials, and it is widely used in bone tissue and other tissue engineering fields [74,75]. For example, Jena et al. made an XG-DA porous network structure scaffold by using XG and dialdehyde alginate (DA) as the raw materials under the cross-linking action of Tetraethyl Orthosilicate (TEOS). The scaffold had good degradability, excellent mechanical properties, and a good protein adsorption effect and thermal stability, and it could effectively promote the formation of an extracellular matrix (ECM), which can be used as a good biological scaffold in the field of tissue engineering [76]. Bernal-Chávez et al. prepared PVA-XG hydrogels using polyvinyl alcohol (PVA) and XG as the base material under the action of three freeze/thaw techniques. The hydrogel showed good cytocompatibility, plasticity, and corrosion resistance, and it can be used in the development and application of biological scaffolds in skin, blood vessels, and other tissue engineering fields, with a broad application prospect (Figure 4B) [71].

#### 2.2.9. Glucan

Glucan is the isotype polysaccharide of a glucose unit connected by glycosidic acid. Depending on the type of glycosidic bond, glucan can be divided into α-glucan and β-glucan. Among them, the research and use of dextran in α-glucan is the most common, and can be used as a substitute for plasma in medicine. Glucan has good biocompatibility and antibacterial effects, and is widely used in the field of tissue engineering [77,78]. Using β-1,3-glucan, chitosan, and biological china, Przekora et al. fabricated a 3D composite scaffold. The scaffold had good elasticity, compression resistance, and showed non-toxicity and biocompatibility. It effectively promoted cell adhesion, growth, and proliferation. Therefore, it can be used as a cell scaffold filler for bone defects, and can be used in bone tissue and other fields [79]. A Dex-G glucose-sensitive hydrogel using dextran and glycidyl methacrylate (GMA) was created via a ring-opening reaction by Yin et al. The hydrogel size could vary according to the concentration of glucose, with good biocompatibility and mechanical properties, and it can be used as a carrier for drug transport in tissue engineering and other fields (Figure 4C) [72].

#### 2.2.10. Heparin

Heparin is named because it was first found in the liver. It is highly acidic, exists in the vascular wall and intestinal mucosa and other tissues, and is a natural anticoagulant substance in animals. Heparin is often used as an anticoagulant in clinical operations or diseases such as hemodialysis, cardiovascular surgery, and myocardial infarction. Heparin can be degraded to produce low molecular weight heparin, and this low molecular weight material is widely used [80,81]. Heparin after 3D printing preparation from biological scaffolds or other composite materials is used for nerve regeneration and vascular tissues, and its application prospect is very broad [82]. For example, a collagen–heparin sulfate biological scaffold was prepared using 3D bioprinting by Jiang et al. The 3D printed composite has good cell compatibility, a good degradation rate, and excellent mechanical properties. At the same time, it could significantly enhance the structural integrity of the cerebral cortex, and it also had a positive role in nerve regeneration and vascular regeneration [83]. An anticoagulant double-tubular biological scaffold was prepared via heat-induced phase separation and electrospinning by Domalik-Pyzik et al. The outer layer of the scaffold was polycaprolactone as the main material, while the inner layer was the polylactide layer modified by heparin. It not only improved the mechanical properties, but it was also confirmed using the endothelial cell culture test that the bilayer scaffold had a positive effect on human aortic endothelial cell lines (HAECs), which can be applied in the field of vascular tissue engineering (Figure 4D) [73].

#### 2.2.11. Fucoidan

**Figure 5:**
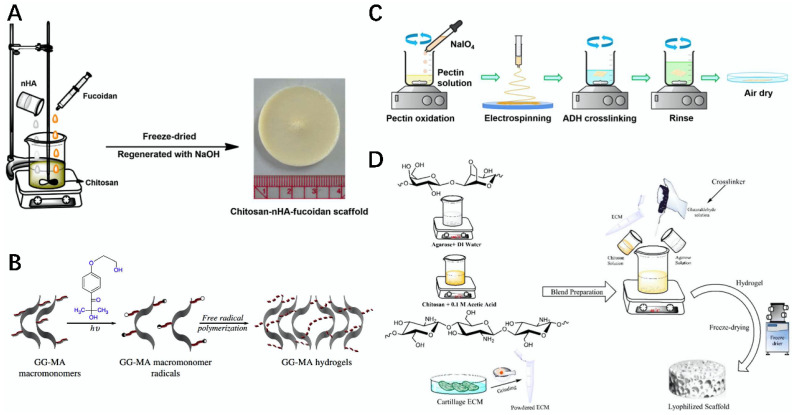
Schematic diagram of the preparation of a composite scaffold and hydrogel using polysaccharides and ECM. (**A**) Schematic diagram of preparation of chitosan–nHA–Fu biocomposite scaffold [86]. (**B**) Schematic diagram of preparation of guar gum–-MA hydrogel [87]. (**C**) Schematic diagram of preparation of pectin hydrogel nanofiber holders [88]. (**D**) Schematic diagram of the preparation of chitosan–SA–ECM scaffold material [89].

Fucoidan (Fu) is a cell wall polysaccharide with a variety of properties such as antiviral, antioxidant, and anti-inflammation. Among them, kelp is a common Fu. Modified or processed Fu can be used in tissue engineering and other fields [84]. For example, Puvaneswary et al. prepared a chitosan–TCP–Fu biological scaffold via freeze-drying with chitosan, tricalcium phosphate (TCP), and Fu as the raw materials, which had a good compressive effect, and it effectively improved the activity of human-derived bone marrow stromal cells and promoted osteogenesis, which can be applied to the field of bone tissue [85]. Lowe et al. prepared a biocompatible chitosan-nHA-Fu biocomposite with chitosan, Fu, and natural nano-hydroxyapatite (nHA) as the materials via freeze-drying. It had the nutrients required by cells and was suitable for cell growth and proliferation, and the scaffold could induce differentiation into bone, and can be applied to bone tissue and other fields (Figure 5A) [86].

#### 2.2.12. Guar Gum

Guar gum is a polysaccharide extracted from the guar bean of the leguminous plant, and its structure is composed of α-D-galactose and β-D-mannopyranose, which usually presents as a white or light brown powder with high solubility, and can be dispersed in water to form a sticky solution, which can be used as a thickening agent and stabilizer. At the same time, it has the effect of controlling blood sugar. Guar gum is processed into bio-ink or composite scaffolds, and can be applied to tissue engineering or other fields through 3D printing [90,91]. For example, Cleymand et al. used guar gum-modified chitosan as a raw material to prepare a guar gum–chitosan bio-ink. The bio-ink had good rheological properties, and the material produced using extruded 3D bioprinting can play an important role in tissue engineering and regenerative medicine [92]. Tiwari et al. first prepared the guar gum–methacrylate (guar gum–MA) macromolecular monomer via the catalysis of 4-dimethyl-amino-pyridine (DMAP) based on guar gum and methacrylates (MA) as raw materials, and then used this monomer as a material under the action of UV light and free radical polymerization and other operations to finally prepare a guar gum –MA hydrogel. The hydrogel exhibited good biodegradability and mechanical properties, with a wide range of raw materials and low preparation difficulty. It is a biomaterial with a broad application prospect, and can be used in tissue engineering and other fields (Figure 5B) [87].

#### 2.2.13. Pectin

Pectin is a kind of natural polysaccharide in the plant cell wall and inner layer; under normal circumstances, it is a white or yellow powder. Its mixture with water is in a ratio of 1:20, and it can form a milky viscous colloidal solution. Pectin has a very good degree of water retention and solubility, and it can help to control blood lipids and blood sugar. As one of the most important polysaccharides, pectin has been widely used in tissue engineering and other fields [93,94]. For example, Kumar et al. used pectin, chitin, and nano-CaCO_3_ as materials, and prepared a pectin–chitin/nano-CaCO_3_ biological scaffold with good biocompatibility, good cell viscosity, and a high cell attachment rate using a freeze-drying method; the degradation and swelling of the scaffold were further controlled, and the cytocompatibility evaluation of human dermal fibroblast (HDF) showed that the scaffold was almost non-toxic to cells, which could be used as a drug delivery carrier in the field of tissue engineering [95]. Li et al. prepared a pectin hydrogel nanofiber scaffold using high phosphate oxidation and electrostatic spinning, and the composite scaffold not only had outstanding mechanical properties and hydrophobicity, but it also had a very good cell adhesion and biocompatibility ability, which can be applied in the field of stem cell tissue (Figure 5C) [88].

#### 2.2.14. Gellan Gum

Gellan gum (GG) is a high molecular linear natural polysaccharide which is inherently resistant to enzymatic degradation and has high stability. After washing in boiling water and then cooling, it forms a hydrogel with excellent firmness. When GG is combined with 3D printing technology, the materials prepared are used in a wide range of applications in the field of brain medicine and tissue [96]. For example, Lozano et al. prepared a bio-ink using peptide-modified RGD-GG, and after 3D printing, prepared a hydrogel which was combined with primary cortical neurons to construct an advanced brain-like structure, a process with low equipment requirements, and which is extremely important for further research into brain injury [97]. Akkineni et al. reported a low acyl GG (LAGG)/high acyl GG (HAGG) blend that was effective in improving the fidelity and cytocompatibility of 3D printing, offering the possibility of customizing tissue engineering materials [98].

### 2.3. Other

Of the natural materials used for 3D printing in addition to proteins and polysaccharides, other natural materials are classified according to other categories in this article. Common other natural materials are decellularized extracellular matrix, extracellular matrix, Matrigel, and peptide-based and DNA bio-inks; these natural materials also play an important role in tissue engineering and other fields.

#### 2.3.1. Decellularized Extracellular Matrix

Decellularized extracellular matrix (dECM) is a kind of natural biological material obtained by removing all cellular components and antigens from animal or human tissues through certain methods. dECM has good mechanical properties, can be implanted in the human body without immune rejection, and can be used to induce cartilage formation and vascular regeneration. Under the functions of the freeze-drying and cross-linking techniques, Yang et al. prepared a porous biological scaffold for dECM that was non-toxic and had good cytocompatibility. At the same time, it could effectively promote bone marrow-derived mesenchymal stem cells (BMSCs) growth, proliferation, and the differentiation of BMSCs, and induce the formation of cartilage tissue, which can be used as an advanced material for cartilage tissue regeneration [99]. Liu et al. modified porcine iliac colorectal cancer cells (PIECs) with bladder acellular matrix (BAM) hydrogel to prepare PIECs/BAM composite gels, which had good biocompatibility and a better effect on promoting neovascularization, and can be used as a material for vascular tissue repair [100].

#### 2.3.2. Extracellular Matrix

Extracellular matrix (ECM) is a complex network of large molecules around cells. ECM contains a large number of signaling molecules, which can be involved in the control of cell growth, shape, and metabolic activities, etc. Additionally, it is also essential for the human body. ECM can be modified to promote bone formation and cartilage repair. For example, Silva et al. used EMC, mesenchymal stem/stromal cell (MSC), and PCL as the original materials, and prepared a PCL–MSC–ECM biological scaffold via fused deposition modeling. The scaffold had good mechanical properties and biological activity, and could effectively promote the expression of osteogenic genes and induce osteogenic differentiation [101]. Garakani et al. used ECM as the main material, modified with chitosan and SA, and prepared a chitosan–SA–ECM scaffold material using a freeze-drying method. After a cytotoxicity test, it was concluded that the scaffold had good cell adhesion. At the same time, the scaffold showed good mechanical properties and biodegradation rate, and is a potential material for cartilage tissue repair (Figure 5D) [89].

#### 2.3.3. Matrigel

Matrigel is an extract derived from EHS tumors. Usually, it is highly insoluble, and part of the solid basement membrane can be used for cell culture. Matrigel can be used in tissue engineering and other fields after being processed into bio-inks or scaffolds [102]. Fan et al. prepared a type of Matrigel-AG hydrogel bio-ink, which had good mechanical properties and cell compatibility, and its properties were very suitable for the rheology of 3D printing. At the same time, the gel showed good rheological properties, acting as a very good biomaterial in 3D printing tissue structure [103]. Schumann et al. modified Matrigel with poly-(L-lactide-co-glycolide) (PLGA) to prepare composite scaffold. The scaffold had excellent mechanical properties, good cell activity, and could accelerate the regeneration of blood vessels. It can be used as a potential material for vascular tissue regeneration [104].

#### 2.3.4. Bio-Inks Based on Peptides and DNA

Peptides are organic compounds containing carboxyl groups and amino groups formed after the dehydration and condensation of amino acids. Peptides are biologically active substances that function in a variety of cells in an organism, and they are essential for the organism. DNA is the abbreviation of deoxyribonucleic acid, which is the genetic information that is required for the synthesis of protein and ribonucleic acid (RNA), which are essential for living organisms. Bio-inks based on peptide and DNA have some good properties that are suitable for 3D printing for tissue engineering [105,106]. For example, Firipis et al. interacted with AG, SA, and peptide to prepare peptide–AG–SA bio-inks. Under the action of 3D printing, the prepared hydrogel composite material had good biological activity and compatibility. It can be used as a promising bio-ink in the field of tissue engineering [107].

## 3. Modification Methods of Natural Biomaterials for 3D Printing

There are many ways to process natural materials for 3D printing. For example, AG and chitosan can be heated to produce a thin film material, which can be used as a substitute for skin tissue [108]. The processing and modification methods of different materials are different, and the suitable processing methods are different. Here, we list the natural materials processed through physical, chemical, and protein self-assembly methods to prepare composite materials and composite scaffolds. These materials play a wide range of applications in tissue engineering, skin engineering, cartilage tissue, bone tissue, and vascular tissue.

### 3.1. Physical Methods

A heating method is a relatively simple processing method for natural materials. The composite materials prepared with natural materials after heating have great application prospects in the field of tissue engineering [109]. For example, Sarasam et al. prepared chitosan–PCL composite membranes using PCL and chitosan as raw materials after heating in a water bath at different temperatures. Not only were the cell activities and mechanical properties of the material improved, but the material also had low cytotoxicity and good biocompatibility, which is suitable for tissue engineering and other fields [110]. Jiang et al. prepared a 3D chitosan/PLAGA composite scaffold using a sintered microsphere technique using chitosan and poly (lactic acid-glycolic acid) (PLAGA) as the materials. The mechanical properties and void size of the scaffold could be changed according to the sintering time and temperature, and could effectively promote the growth and proliferation of MC3T3-E1 osteoblasts, which have broad application prospects in the field of bone tissue [111].

**Figure 6:**
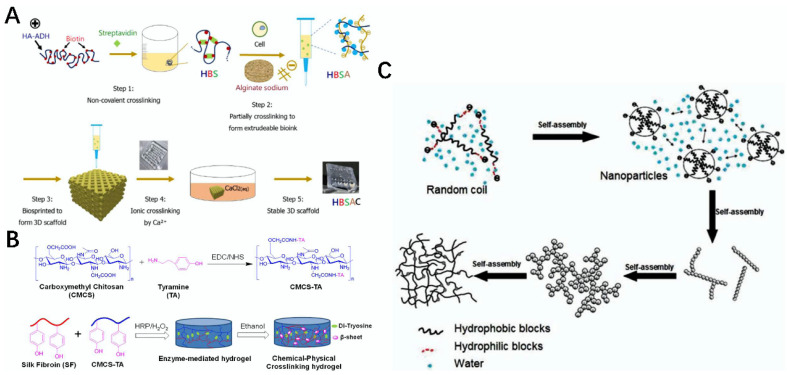
Processing and modification of natural biological materials. (**A**) Schematic diagram of HBSAC hydrogel scaffold prepared via Ca^2+^ modification [113]. (**B**) Schematic diagram of the preparation of CMCS-TA hydrogels under HRP operation [114]. (**C**) Schematic diagram of the protein self-assembly of silk fibroin in aqueous solution [115].

Some natural materials are suitable for physical processing and modification, and these composite materials can exhibit better properties. Among them, Ca^2+^ modification is a common natural material processing method. For example, Kim et al. prepared alginate–SF bio-inks by modifying alginate and SF with Ca^2+^. With the help of 3D printing technology, an alginate–SF hydrogel has been printed. The hydrogel had good bioactivity and cytocompatibility, and can be used as an advanced material for tissue engineering [112]. Nedunchezian et al. first prepared a HA–biotin–streptavidin (HBS) hydrogel with HA–biotin (HAB) and streptavidin, and then modified the HBS hydrogel with sodium alginate. Under the action of a 3D bio-printer, HBSA biological scaffolds were prepared. Finally, HBSAC (HBSA + Ca^2+^) hydrogel scaffolds were obtained after modification with CaCl_2_ solution. The hydrogel scaffolds not only had good biocompatibility, but also could induce the chondrogenic differentiation of adipose-derived stem cells (ADSCs). They had a positive effect on cartilage growth and formation (Figure 6A) [113].

### 3.2. Chemical Modifications

Bio-inks made from chemically modified natural materials are used for 3D bioprinting and have important applications in tissue engineering. For example, Petta et al. prepared HA–Tyr bio-ink by modifying HA with Tyramine (Tyr) under the action of photo-cross-linking and enzymatic reaction. The ink was simple to prepare and had good biocompatibility, and can be used as a potential material for 3D printing tissue engineering materials [116]. Li et al. prepared a CMCS-TA composite hydrogel by cross-linking carboxymethyl chitosan (CMCS) and SF raw materials with hydrogen peroxide and horseradish peroxidase (HRP). The hydrogel was implanted subcutaneously in mice. There was no inflammatory reaction to the gel, showing good biocompatibility. At the same time, the gel had the characteristics of good biocompatibility and fast degradation rate, and is an advanced biomaterial suitable for cartilage tissue (Figure 6B) [114].

### 3.3. Protein Self-Assembly

Protein self-assembly is a widely used natural material processing method, which can be used to process natural materials into bio-ink, which is suitable for tissue engineering and other fields [117]. For example, Zhang et al. prepared an HA–fibrin hydrogel by combining thrombin, thiol-modified HA, 2-dithiopyridyl-modified HA, and fibrinogen, which could effectively promote cell growth and proliferation. A new strategy for tissue engineering was proposed in [118]. Ming et al. used Silk I as a raw material to prepare a silk membrane using a protein self-assembly method in aqueous solution, which was not only thermally stable, but also showed positive effects in biocompatibility, and has broad application prospects in the field of tissue engineering (Figure 6C) [115].

## 4. Method of 3D Printing and Its Introduction

Three-dimensional printing, also known as additive manufacturing, is a type of rapid prototyping technology that uses adhesive materials such as powdered metal or plastic to construct objects layer-by-layer on the basis of digital model files [119]. According to the American Society for Testing and Materials (ASTM) classification standards, 3D printing technology can be divided into seven categories: powder bed fusion molding technology (PBF), material extrusion molding technology (ME), binder injection molding technology (BJ), photopolymerization curing technology, material inkjet forming technology (MJ), direct energy deposition technology (DED), and sheet lamination technology (SL) [120]. Currently, the commonly used 3D bioprinting technologies mainly include extrusion bioprinting, inkjet bioprinting, laser-assisted bioprinting, fusion deposition modeling (FDM) and direct ink writing (DIW) [121]. These common 3D printing methods and their comparison are listed in Table 1.

### 4.1. Extrusion Printing

**Figure 7:**
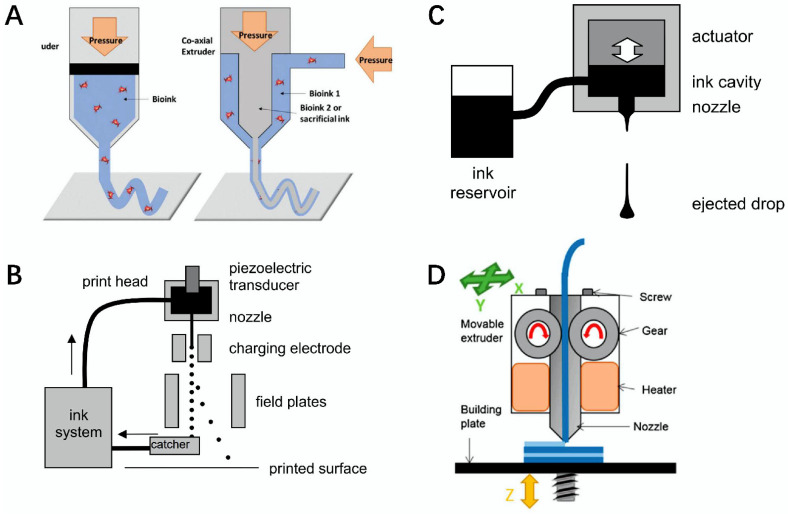
Schematic diagram of 3D printing methods and principles. (**A**) Extrusion and coaxial extrusion [122]. (**B**) Basic principle of continuous inkjet [123]. (**C**) Schematic diagram of an on-demand inkjet printer [123]. (**D**) Schematic diagram of 3D printing mechanism of molten deposition modeling [124].

Extrusion printing is one of the most common techniques in 3D printing. The bio-ink conveys the ribbon through the nozzle or tip, and then it obtains the desired layer of the shape via the x/y movement of the print head. After the first layer is printed, the nozzle is moved up or the receiving plate is moved down to start printing the second layer, and so on. Compared with other types of bioprinting, extruded 3D printing is more compatible. It can continuously extrude bio-ink and produce products with structural integrity in a rapid manufacturing process, which results in 3D printing with fewer defects and reduced complexity (Figure 7A) [122]. Extrusion-based printing can reach a 200 mm resolution, which is lower than laser or inkjet-based systems, but the technology is fast to manufacture and can generate anatomically shaped structures [123,124]. In the print head or collection phase, extruded bioprinting systems rely on distributing the large polymer or hydrogel chains with micropores and locating them through computer-controlled movements [125,126].

### 4.2. Inkjet Printing

Inkjet printing is one of the pioneers of 3D printing technology, with the advantages of low cost, high efficiency, high precision, etc. The purpose of this is to better achieve cell printing to manufacture tissues or organs [127]. Inkjet printing is mainly divided into two modes: continuous inkjet and on-demand drip ink. In continuous inkjet printing, ink flows out of nozzles or holes under controlled pressure and forms a column. Due to the Rayleigh–Plateau instability of the fluid, the column liquid splits into individual droplets. Whether droplets are needed or not, continuous inkjet devices will produce droplets, which are prone to waste and the pollution of raw materials (Figure 7B,C) [123].

### 4.3. Laser-Assisted Printing

Laser-assisted printing is a phenomenon based on light-induced forward transfer. The laser beam is focused on a slide covered with a thin layer of gold, on which the bio-ink is spread. As the laser pulse creates more pressure between the gold layer, a bubble is created inside the bio-ink layer, pushing the underlying cells toward the collector slider [128]. The advantage of laser assistance is that it does not require direct contact with the printer to avoid mechanical stress. In addition, it has the advantage of high resolution and precision, but it may also have some negative effects, such as the easy exposure of cells to ultraviolet light during the printing process [129,130].

### 4.4. Fused Deposition Modeling

Fused deposition modeling (FDM) is an extrusion-based 3D printing method in which the material is extruded from the nozzle and diffuses in subsequent layers onto the construction plate. FDM 3D printing technology has the advantages of accurate quantity, the fusion of different filling densities, environmental protection, etc., but its main disadvantages are the use of solvents and low drug loading [131]. In FDM 3D printing, the filament fed into the corresponding nozzle is heated to a molten state and is deposited layer by layer. After the anterior layer is formed, another layer of melted filament will be deposited on it until the printing is complete (Figure 7D) [124,132].

### 4.5. Direct Ink Writing

Direct ink writing (DIW) is a multimaterial and multifunctional manufacturing method that shows great potential in the fields of structural materials, tissue engineering, and soft robotics. During the DIW process, viscoelastic ink is extruded from the nozzle of the DIW 3D printer as a print fiber, which is deposited to form a pattern as the nozzle moves [133]. DIW 3D printers typically have two key components; one is to have multiple extrusion heads, and the other is to have the xyz stage to control the deposition path of the print head. The advantage of DIW inks is their ability to heal quickly after extrusion [134].

## 5. Application

The natural materials used for 3D printing have good stability, compatibility, and degradability [135]. They are processed and modified via various methods to make bio-inks, and then under the action of 3D printing technology, the prepared composite materials or composite scaffolds can have important applications in the fields of tissue engineering, skin engineering, cartilage tissue, bone tissue, and vascular tissue [136,137]. These natural materials have different properties after being processed and modified, and can be used in different fields according to their different properties.

### 5.1. Tissue Engineering

Tissue engineering is a vast field, and certain natural materials that are used for 3D printing have been modified and prepared as composite nanoparticles or bionic scaffold materials, which are stable and have high cellular activity, and can be used as advanced materials in the field of tissue engineering [138,139]. For example, Sumathra et al. prepared an HAP–pectin composite nanoparticle using hydroxyapatite (HAP) and pectin as the materials; it not only had outstanding biocompatibility, but it also had good bioactivity and mechanical properties, and promising applications in the field of tissue engineering [140]. Kakkar et al. prepared a KE–chitosan–gelatin composite nanoparticle using gelatin and KE via a freeze-drying method. The scaffold exhibited good thermal stability, porosity, and mechanical properties, while the scaffold showed good cellular activity and could promote the proliferation and differentiation of relevant cells, which could be used as a potential material for tissue engineering [141].

**Figure 8:**
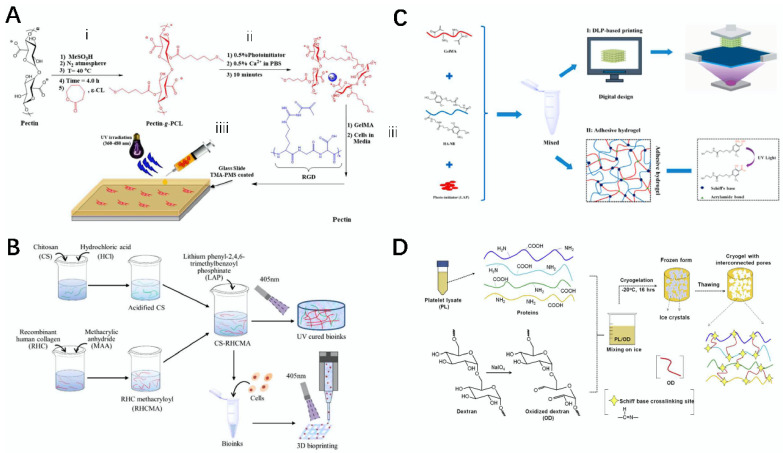
Schematic diagram of hydrogels, bio-scaffolds, and bio-inks prepared from related natural materials and used for 3D printing. (**A**) Schematic diagram of the preparation of GelMA/pectin-g-PCL hydrogels [143]. (**B**) Preparation of CS–RHCMA bio-ink and its use for 3D printing [144]. (**C**) Schematic diagram of the preparation of 3D printed GelMA/HA-NB/LAP hydrogel [145]. (**D**) Schematic diagram of the preparation of PO/OD bio-scaffold [146].

The design and preparation of new hydrogels and bio-inks are important for tissue engineering, as hydrogels are inherently sustainable for drug release and are hydrophilic, while the use of bio-inks in 3D printing produces stable composites, both of which are advanced materials in the field of tissue engineering [142]. For example, Fares et al. first prepared pectin grafted polycaprolactone (Pectin-g-PCL) from pectin, and then modified it with Ca^2+^, GelMA, and arginine-glycine-aspartic acid (RGD) to produce GelMA/pectin-g-PCL hydrogels. The hydrogel not only showed good adjustability, unique mechanical properties, and excellent stability, but it also had good cytocompatibility and stability, and the material was simple to prepare and could promote the growth of osteoblasts in vitro, which can be used as a potential material in tissue engineering and regenerative medicine, and has a very promising application (Figure 8A) [143]. Yang et al. prepared RHC methacryloyl (RHCMA) by modifying recombinant human collagen (RHC) with methacrylic anhydride, and then modified RHCMA with acidified chitosan to produce a CS–RHCMA bio-ink that had adjustable degradability and mechanical properties, as well as good biocompatibility, and could be used for the construction of various tissue engineering materials using extrusion bioprinting (Figure 8B) [144].

### 5.2. Skin Engineering

The skin is the most direct contact tissue between the human body and the outside world, and it plays a very important role in protecting the health of the human body. However, skin trauma is often unavoidable, and the repair of skin trauma involves three stages: inflammation, proliferation, and maturation [147]. How to repair skin wounds more effectively is also the urgent hope of researchers. There are many common skin wound repair methods. Among them, the use of polysaccharide, a natural material, to prepare hydrogels is a good method for skin tissue wound repair [148]. For example, Cao et al. used human-like collagen (HLC) and carboxymethylated chitosan (CCS) as the original materials, and prepared a hydrogel with good histocompatibility, good mechanical properties, high porosity, and high pore size via enzyme-chemical double cross-linking technology. The HLC-CCS hydrogel bioscaffold can be used to mimic the three-dimensional network structure of the human extracellular matrix, provide cells with the required attachment points and nutrients, and promote the expression of vascular endothelial growth factor (VEGF). The hydrogel scaffold showed a good ability to regenerate skin wounds, and can be used for skin wound repair [149].

The combination of 3D bioprinting technology and natural materials has been used to prepare composite materials that can be widely used in the repair of skin tissue [150]. For example, Intini et al. prepared a 3D chitosan polymer scaffold with chitosan as the main raw material, using extrusion bioprinting technology, which had high cellular activity, good biocompatibility, can be used for cell line culture, can be used for wound repair in diabetic rats, and can effectively promote the growth of human skin cells [151]. Zhou et al. used GelMA and butanamide-linked hyaluronic acid (HA-NB) as the raw materials, and lithium phenyl-2,4,6-trimethylbenzoylphosphinate (LAP) as a cross-linking agent to prepare a GelMA/HA-NB/LAP bio-ink. The ink was made into a hydrogel under the action of the 3D printing technology of DLP. The composite hydrogel had good cell viscosity and biocompatibility, which can effectively promote angiogenesis and accelerate skin regeneration. It can be applied in the field of skin tissue (Figure 8C) [145].

### 5.3. Cartilaginous Tissue

Cartilage tissue is a very important tissue in the body, and plays a very important role in the human body. However, the damage or loss of cartilage tissue is also a condition that people have to deal with. Cartilage has a low ability to repair itself, and is generally difficult to heal. Articular cartilage loss is one of the most common types of cartilage tissue loss [152]. Damaged or absent articular cartilage can be very damaging to patients, but by using natural materials such as dextran, composite scaffolds have been prepared that can be used in the treatment of patients with joint defects [153]. For example, Kuo et al. first prepared a chitosan/γ-poly (glutamic acid) (γ-PGA) bio-scaffold using a freeze-drying method, and after modification with human serum albumin (HSA), poly-L-lysine (PLL) and elastin, the scaffold could effectively promote the proliferation and differentiation of chondrocytes as a potential material for cartilage repair [154]. Şeker et al. prepared a PL/OD bioscaffold with PL and oxidized dextran (OA) as the materials. The composite scaffold had good biodegradability, cytocompatibility, and low toxicity, and human adipose stem cells (hASCs) still maintained good cell activity 7 days after inoculation. It promoted chondrogenesis and can be used as a potential material in the field of cartilage and other tissue engineering (Figure 8D) [146].

**Figure 9:**
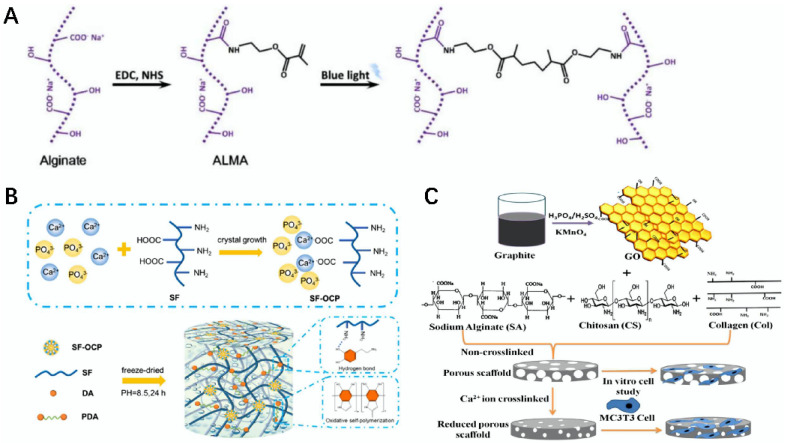
Schematic diagram of biomodification and preparation of bio-scaffolds using related natural materials. (**A**) Schematic diagram of the modification of ALMA using alginate [158]. (**B**) Schematic diagram of the preparation of SF/OCP/PDA scaffold [159]. (**C**) Schematic diagram of the preparation of SA–Chitosan–Col–GO composite scaffold [160].

The natural materials used for 3D printing were modified to prepare bio-inks, and the composites prepared under the action of 3D bioprinting technology can be widely used for cartilage tissue repair [155,156]. For example, Lee et al. prepared a Col–OPCs–OHA composite scaffold using Col, oligomeric proanthocyanidin (OPCs), and oxidized hyaluronic acid (OHA) as materials in the presence of 3D printing technology, which not only had excellent mechanical properties, degradability, and low toxicity, but also had good biocompatibility and cellular activity, which can be used within the field of articular cartilage repair [157]. Cao et al. modified methacrylated alginate (ALMA) with alginate, modified with PCL, and then prepared a PCL–ALMA composite scaffold via 3D printing technology. This material scaffold can effectively promote chondrocyte proliferation and improve cartilage differentiation, which can provide a new strategy for cartilage regeneration (Figure 9A) [158].

### 5.4. Bone Tissue

Some people have diseases such as osteoporosis and bone defects, which can be extremely harmful for patients. Therefore, the development of bionic bone tissue engineering scaffolds is of particular importance and can achieve a very good effect if relevant natural materials are combined with the development of bone scaffolds [161,162]. For example, Peng et al. used SF, polydopamine (PDA), and octacalcium phosphate (OCP) as materials to prepare a SF/OCP/PDA three-dimensional network bio-scaffold using a freeze-drying method, which showed more positive compressive capacity and better biocompatibility, and the scaffold could effectively accelerate the phospholime deposition rate, which can be used as a potential material for bone defect repair (Figure 9B) [159]. Kolanthai et al. first prepared a SA–chitosan–Col scaffold using a freeze-drying method, and after modification by graphene oxide (GO), an SA–chitosan–Col-GO biocomposite scaffold was made, which possessed stable mechanical properties and good biocompatibility, and the growth and proliferation effects of the mouse osteoblasts were promoted on this scaffold, which could be used to promote bone tissue regeneration (Figure 9C) [160].

**Figure 10:**
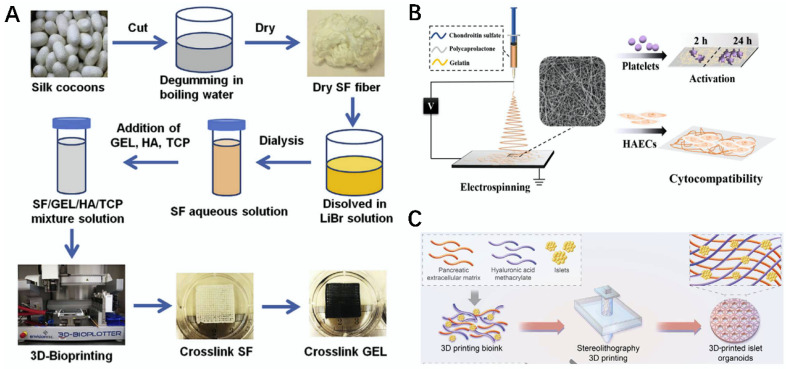
Schematic representation of the preparation of bio-scaffolds, nanofibers, and hydrogels using related natural materials. (**A**) Schematic diagram of SF scaffold prepared via 3D printing technology [166]. (**B**) Schematic representation of the preparation of CS–gelatin–PCL composite nanofibers [167]. (**C**) Schematic diagram of a hydrogel printed using HAMA/pECM bio-ink [168].

The natural materials used for 3D printing have been interacted with other materials to prepare bio-inks, and the composites prepared via 3D bioprinting technology have been used for good applications in the field of bone tissue [163,164]. For example, Iglesias-Mejuto et al. used 3D printing to prepare SA–HAP hydrogel scaffolds, which were biocompatible and non-toxic, to promote the proliferation and differentiation of MSCs, as well as the movement of fibroblasts towards damaged areas, providing a new strategy for bone regeneration [165]. Wei et al. prepared a silk protein composite bio-ink using gelatin, HA, tricalcium phosphate (TCP), and SF as the materials, prepared a silk protein scaffold via 3D printing, and then modified the scaffold using human platelet-rich plasma (PRP). This material effectively promoted the growth and proliferation of human adipose derived mesenchymal stem cells (HADMSC), providing a new and effective method for osteogenic differentiation (Figure 10A) [166].

### 5.5. Vascular Tissue

The number of victims of vascular diseases is increasing worldwide, increasing the need for vascular tissue substitutes. Intimal hyperplasia and thrombosis are the primary problems of vascular tissue engineering transplantation. Heparin is a natural polysaccharide that is effective in preventing thrombosis [169]. Currently, there are various treatments for vascular diseases, among which the composite materials prepared by modifying natural materials are widely used [170]. For example, Liu et al. prepared an alginate/gelatin-based hydrogel with a soy protein/peptide biomaterial scaffold using sodium alginate, gelatin, soy protein, and soy peptide powder as the raw materials. The composite scaffold had excellent mechanical properties and good biocompatibility, and effectively promoted the growth and proliferation of cells, while the scaffold effectively promoted the growth of new blood vessels and has broad applications in vascular repair [171]. Kong et al. prepared a CS–gelatin–PCL composite nanofiber by combining CS, gelatin, and polycaprolactone (PCL) under the action of electrostatic spinning, which could change the diameter and morphology of the fibers by changing the percentage of CS, while the composite nanofibers fibers with characteristics such as high porosity and cell adhesion very effectively promoted tissue repair and the regeneration of blood vessels, and has promising applications in vascular tissues (Figure 10B) [167].

Bio-inks have been prepared using natural materials modified with other materials, and the composites prepared via the action of 3D bioprinting technology offer new strategies for vascular tissue-based diseases [172,173]. For example, Wang et al. prepared a HAMA/pECM bio-ink using hyaluronic acid methacrylate (HAMA) and pancreatic extracellular matrix (pECM), and under printing via 3D printing technology, a HAMA/pECM hydrogel was prepared, which not only effectively maintained the stability of blood glucose levels, but also promoted the ability of neovascularization to attach and grow, contributing to the formation of vascular networks (Figure 10C) [168].

## 6. Conclusions

For each of the natural materials mentioned in this article, we now present a small summary of their latest applications in the field of 3D printing. The composite hydrogels and various biomaterials obtained from the preparation of bio-inks using PL, Col, SF and chitosan, after 3D printing, can be used in the construction and repair of biological tissues and organs in vitro. Three-dimensional printed bio-scaffolds made of AG, gelatin and KE have good rheology and can be used to repair wounds and promote vascular regeneration. Natural polysaccharides such as ECM, Fu, SA and pectin are prepared as bio-ink and then 3D printed to produce composites and bio-scaffolds with functions such as inducing bone differentiation and promoting bone formation, which are suitable for bone tissue applications. Materials or scaffolds of Col, CS, HA, albumin and fibronectin, prepared by the action of 3D printing, have the function of inducing the proliferation and differentiation of related chondrocytes, which are very suitable for cartilage tissue regeneration. Materials such as hydrogels prepared by Laminarin bio-inks by the action of 3D printing can be used for the sustainable culture of cells. Heparin, after 3D bioprinting, can be used in nerve regeneration and vascular tissues, and the bio-scaffolds and composites prepared are very promising. Natural polysaccharides based on materials such as Matrigel, HA, dextran, CRG, XG and peptide and DNA-based bio-inks are processed to produce composites that play other important roles in tissue engineering and other fields.

In conclusion, natural materials for 3D printing are rich in variety and diverse in nature, and they can be used as a class of promising advanced materials with certain stability, cell adhesion, and biocompatibility, which can be processed and modified to prepare bio-inks. They can then used for 3D bioprinting to prepare composite scaffolds or composites that play an increasingly important role in various fields such as tissue engineering and regenerative medicine. However, there are still some limitations regarding bio-inks and printing methods. The biocompatibilities and mechanical properties of some natural materials need to be improved, some properties of related bio-inks still need to be improved, and the development of some advanced bioprinters needs to be accelerated. The use of related materials such as hydrogels and scaffolds still needs to be validated in clinical trials. Therefore, research into the use of natural materials for 3D bioprinting needs to be further developed.

## Figures and Tables

**Table 1:** Common methods of 3D printing and their comparison.

Printing Methods	Security	Technical Difficulties	Cost	Print Speed	Advantage
Extrusion printing	Safety	Lower	Lower	Faster	Strong compatibility
Inkjet printing	Safety	Lower	Lower	Slower	High precision
Laser-assisted printing	Safety	Higher	Higher	Faster	High resolution
Fused deposition modeling	Safety	Lower	Lower	Slower	Environmental protection
Direct ink writing	Safety	Lower	Lower	Faster	Rapid self-healing ability

## Data Availability

The data presented in this study are available in the article.
